# Late-onset and relapsed cytokine release syndrome after nivolumab treatment in a patient with head and neck squamous cell carcinoma: a case report

**DOI:** 10.3389/fonc.2025.1508682

**Published:** 2025-02-07

**Authors:** Tomoyuki Otsuka, Yoshiki Kojitani, Fumio Imamura, Junko Fukutake, Minako Nishio, Takashi Fujii, Toshihiro Kudo

**Affiliations:** ^1^ Department of Medical Oncology, Osaka International Cancer Institute, Osaka, Japan; ^2^ Department of Head and Neck Surgery, Osaka International Cancer Institute, Osaka, Japan

**Keywords:** immune checkpoint inhibitor, immune-related adverse event, cytokine release syndrome, head and neck squamous cell carcinoma, case report

## Abstract

Nivolumab, an anti-programmed death-1 (PD-1) receptor monoclonal antibody, has proven effective in treating platinum-resistant metastatic head and neck squamous cell carcinoma. Immune-related adverse events (irAEs) are well-known complications of PD-1 inhibitors. Meanwhile, cytokine release syndrome (CRS), a life-threatening immune-related adverse event, rarely develops due to nivolumab monotherapy. Here, we report a case of a 65-year-old man with squamous cell head and neck carcinoma of an occult primary origin who developed nivolumab-associated late-onset CRS that recurred. The patient was admitted with symptoms of fatigue, fever, hypotension, and respiratory distress. The diagnosis of CRS was supported by the elevated serum levels of interleukin-6 and ferritin, and the patient responded well to high-dose methylprednisolone. CRS recurred during steroid tapering, coinciding with an increased tumor burden; however, it was successfully managed with increased steroid dosing. Early detection and treatment with steroids are essential for the management of CRS.

## 1 Introduction

Immune checkpoint inhibitor (ICI) therapy has become the standard treatment for patients with metastatic head and neck squamous cell carcinoma (1). However, ICI therapy is associated with various immune-related adverse events (irAEs) including cytokine release syndrome (CRS). Although CRS rarely develops with ICI monotherapy, it is a potentially fatal complication (2). Here, we report a case of a patient with squamous cell head and neck carcinoma of an occult primary origin who experienced repeated recurrence of ICI-induced CRS.

## 2 Case description

**Figure 1:**
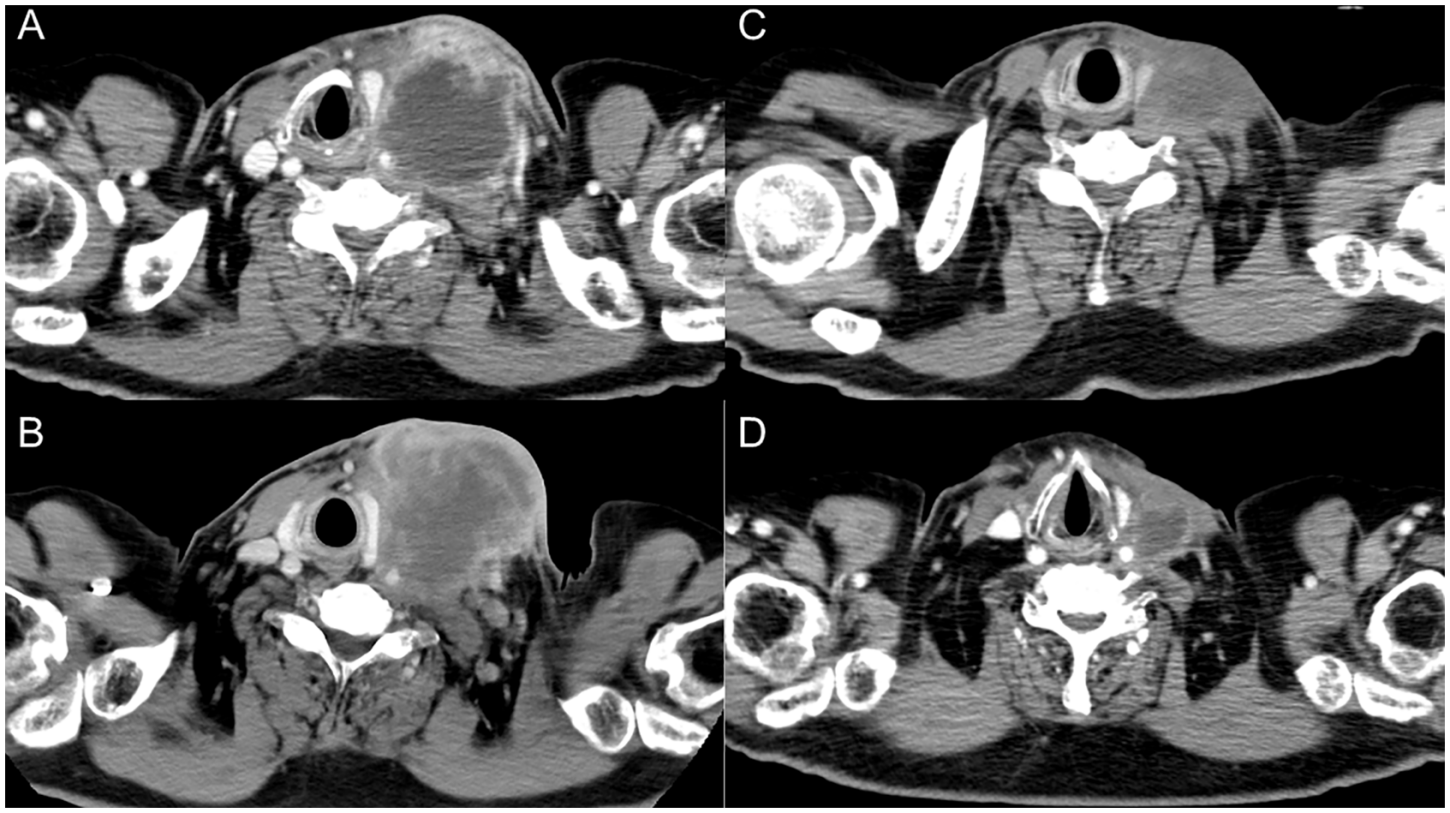
Serial images of the left cervical lymph node. **(A)** At initial diagnosis. **(B)** After three cycles of nivolumab therapy. **(C)** After six doses of paclitaxel and cetuximab. **(D)** Two months after steroid therapy.

A 65-year-old man, a 90-pack-year smoker, presented with swelling of the left cervical lymph nodes. Histological examination of the excised lymph node confirmed squamous cell carcinoma. Immunostaining for p16 and *in situ* hybridization of Epstein-Barr virus-encoded RNA yielded negative results. Whole-body computed tomography (CT), magnetic resonance imaging of the cervical region, positron emission tomography, and upper gastrointestinal endoscopy were performed; however, no primary tumor was identified. The patient was diagnosed with squamous cell head and neck carcinoma with an occult primary origin, classified as clinical T0N3bM0, stage IVB with programmed cell death-ligand 1 expression (combined positive score of 80) (**Figure 1A**).

The patient was treated with cisplatin 60 mg/m^2^ and docetaxel 60 mg/m^2^, both on day 1 every 3 weeks in two cycles, and 5-fluorouracil 700 mg/m²/day on days 1 to 4 every 3 weeks in two cycles. The left cervical lymph node metastasis was enlarged, leading to the determination that radical treatment was not feasible. The patient was treated with nivolumab 480 mg/body alone on day 1 every 4 weeks for three cycles. The lymph node metastatic lesions further increased in size, resulting in skin invasion (**Figure 1B**). Treatment was switched to weekly paclitaxel and cetuximab (paclitaxel 80 mg/m²; cetuximab first course of 400 mg/m² followed by 250 mg/m²).

**Figure 2:**
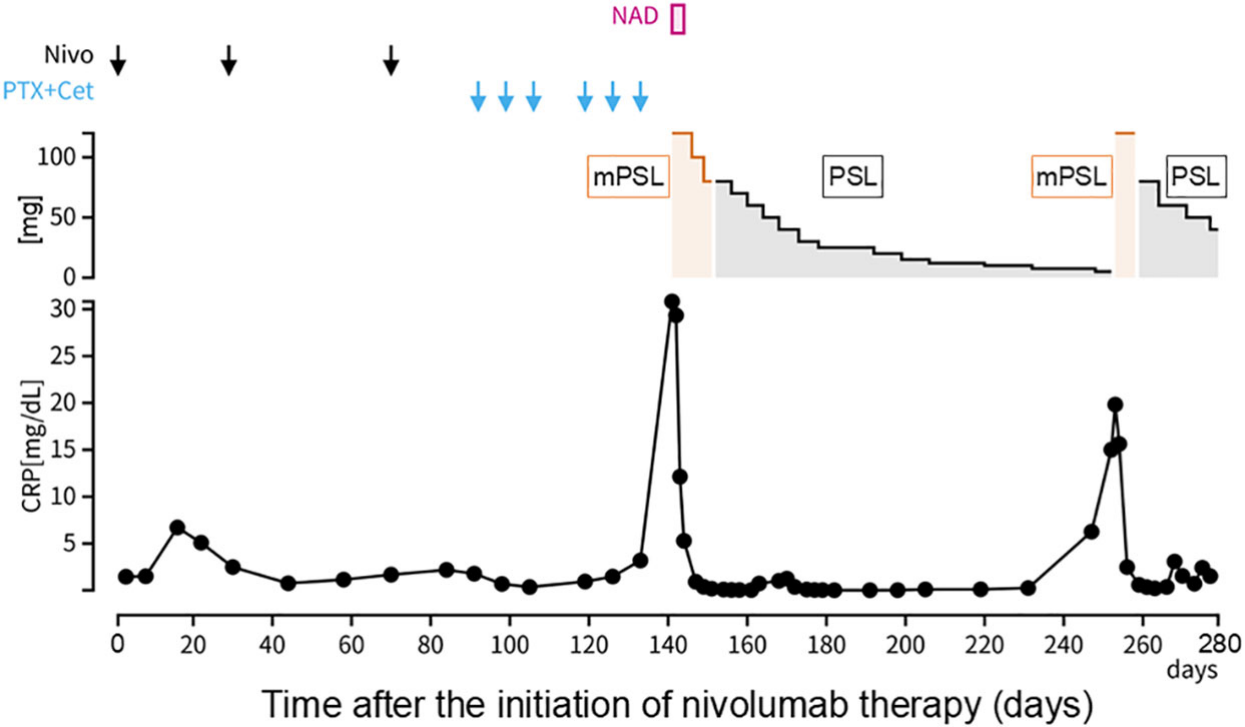
Clinical course. Day 1 was defined as the day on which nivolumab was first administered. CRP, C-reactive protein; Nivo, nivolumab; PTX, paclitaxel; Cet, cetuximab; NAD, noradrenaline; mPSL, methylprednisolone; PSL, prednisolone.

Approximately 50 days after the first administration of paclitaxel and cetuximab, the patient was hospitalized because of progressive fatigue and fever for more than 3 days. On admission, he had a headache, a fever of 38.8°C, and oxygen saturation of 93% while receiving oxygen at 5 L/min. The other vital signs were as follows: blood pressure, 114/87 mmHg; heart rate, 120 beats/min; and respiratory rate, 22 breaths/min. The laboratory findings included a CRP level of 30.8 mg/dL, a white blood cell count of 6,050/μL, an absolute neutrophil count of 2,541/μL, an absolute lymphocyte count of 2,916/μL, hemoglobin at 13.9 g/dL, a platelet count of 213,000/μL, and a creatinine level of 1.66 mg/dL. CT revealed ground-glass opacities in both lungs and shrinkage of the left cervical lymph node metastasis (**Figure 1C**). Five hours after admission, the patient’s blood pressure decreased to 76/46 mmHg. Although approximately two liters of normal saline were rapidly infused intravenously, the patient remained hypotensive. The patient’s blood pressure was maintained within the normal range by continuous intravenous administration of norepinephrine (0.025 mg/kg/min). Because sepsis could not be ruled out from the clinical course, the patient was empirically treated with broad-spectrum antibiotics. However, no infectious organisms were detected in sputum, urine, and peripheral blood cultures. On admission, the serum interleukin-6 (IL-6) and ferritin levels were found to be elevated (420 pg/mL and 1150 ng/mL, respectively). Under the suspicion of irAEs, including CRS, the patient was started on intravenous methylprednisolone (2 mg/kg daily). Three days after initiating methylprednisolone administration, blood pressure was maintained without norepinephrine use. Additionally, renal function improved and creatinine level decreased to 0.85 mg/dL. Intravenous methylprednisolone was switched to oral prednisolone 1 mg/kg, and the dose was tapered over the subsequent 2 months (**Figure 2**). Furthermore, the interstitial shadows in the lungs improved and the left cervical lymph node metastasis shrank (**Figure 1D**). Paclitaxel and cetuximab were not restarted.

During the course of tapering prednisolone from 7.5 mg/day to 5 mg/day in the outpatient setting, the patient experienced a recurrence of a fever of 38.5°C and was readmitted. CT performed on readmission showed increased left cervical lymph node metastasis and new metastases to the mediastinal and right hilar lymph nodes. The serum IL-6 and ferritin levels were again elevated (97.4 pg/mL and 567.1 ng/mL, respectively), suggesting a relapse of CRS. Methylprednisolone 2 mg/kg was administered intravenously. Two days after the reinitiation of methylprednisolone, the patient’s fever resolved. Intravenous methylprednisolone was switched to oral prednisolone (1 mg/kg), and the dose was tapered slowly again without fever recurrence (**Figure 2**). The patient’s general condition was poor, and the patient preferred the best supportive care. Eight months after the discontinuation of nivolumab, he died of cancer progression.

## 3 Discussion

We present a case of a male patient who developed CRS 2 months after cessation of nivolumab therapy. The patient presented with high fever and hypotension, along with an elevated C-reactive protein level, initially suggesting the possibility of septic shock. However, all bacterial cultures, including those of sputum, urine, and blood, were negative. Elevated serum IL-6 and ferritin levels supported the diagnosis of nivolumab-induced CRS (3). CRS has been reported to cause multi-organ failure (4, 5). In this case, respiratory impairment, renal failure, and headache considered a neurological symptom, were observed. The patient’s condition improved with high-dose steroid therapy. Distinguishing CRS from sepsis can be challenging in clinical settings. Ferritin levels tend to be higher in patients with CRS than in those with sepsis (4). It has been reported that ferritin levels increase during the onset of irAEs, which may facilitate their diagnosis (6). Elevated ferritin levels may help diagnose CRS as an irAE over sepsis. Furthermore, interferon-gamma is not expected to increase in sepsis, which suggests that it may help differentiate between CRS and sepsis (4).

ICI-induced CRS typically manifests within 1 month of ICI initiation (2, 5). However, in the case of our patient, CRS developed 2 months after the final cycle of nivolumab and during the post-treatment phase. The mechanisms underlying delayed-onset CRS following ICI monotherapy are not well understood but may involve prolonged immune activation due to residual T-cell stimulation (7). While nivolumab was ineffective, subsequent treatment with paclitaxel and cetuximab resulted in a dramatic therapeutic response. This response may have triggered the release of tumor antigens, activating immune cells and initiating an immune response (8). Therefore, the initial CRS may have been influenced by neoantigen release during the post-treatment phase.

The patient’s condition initially improved after the administration of intravenous methylprednisolone. However, CRS flare-ups coincided with tumor progression. High tumor burden is reportedly associated with irAE severity (9). In this case, the increase in tumor volume may have contributed to CRS recurrence. During steroid treatment for CRS recurrence, the cancer continued to progress. Further research is warranted to develop strategies for managing CRS to allow early resumption of chemotherapy.

CRS has rarely been reported in association with nivolumab monotherapy (2). This syndrome, which manifests as sepsis-like symptoms, may be overlooked because of its rapid progression and presents a diagnostic challenge (10). Consequently, CRS caused by ICI monotherapy may be underreported and easily missed. To monitor CRS risk following ICI, clinicians should conduct routine follow-ups to assess clinical symptoms such as fever and hypotension (11). Clinicians should consider both CRS and sepsis in patients previously treated with ICIs who present with fever and hypotension and promptly administer steroids alongside antimicrobial therapy. If the condition does not improve with steroids, tocilizumab, an anti-human IL-6 monoclonal antibody, is recommended to manage CRS (12).

In conclusion, our patient who developed late-onset CRS that recurred after nivolumab therapy initially responded well to steroid treatment. Clinicians must remain aware of the possibility of CRS occurring even after the cessation of ICI therapy.

## Data Availability

The original contributions presented in the study are included in the article/supplementary material, further inquiries can be directed to the corresponding author/s.
